# Can Mediterranean Diet Have a Positive Impact on Kidney Health? A Pending Answer to a Long-Time Question

**DOI:** 10.3390/nu14204366

**Published:** 2022-10-18

**Authors:** Lara Caldiroli, Paolo Molinari, Matteo Abinti, Chiara Rusconi, Giuseppe Castellano, Simone Vettoretti

**Affiliations:** 1 Unit of Nephrology, Dialysis and Kidney Transplantation, Fondazione IRCCS Ca’ Granda Ospedale Maggiore Policlinico di Milano, 20122 Milan, Italy; 2 Department of Clinical Sciences and Community Health, Università degli Studi di Milano, 20122 Milan, Italy

**Keywords:** med diet, chronic kidney disease, nutrition, prevention

## Abstract

Dietary studies conducted in chronic kidney disease (CKD) patients were focused on the quantities of single nutrients, however it is possible that the excessive attention put on the restriction of proteins, sodium, potassium and phosphorus may compromise the overall quality of the diet in terms of micronutrients and palatability. Instead, concentrating on the nutritional quality healthy dietary patterns, may provide a better approach to improve nutritional prescriptions in CKD patients. All these dietary regimens share common features as reduced content of red meat, salt and saturated fatty acids, and higher fiber content, but may differ in terms of single nutrients consumption. In particular, Mediterranean Diet (Med Diet) has been associated with reduced incidence of diabetes, cardiovascular diseases and obesity, all conditions that are also strictly related to CKD. Given its low content of animal proteins and high contents of fiber it is possible that Med Diet may exert also positive effects on CKD as well as on its metabolic complications. In this review we summarize the role of Med Diet in primary prevention of CKD and on its progression.

## 1. Introduction

Chronic kidney disease (CKD) is a worldwide health problem that affects about 10% of the general adult population [1,2,3]. This disease has global implications, ranking as the 17th leading cause of life-expectancy loss in 2015 [4]. In Italy the prevalence of this disease assesses around 8%, with little inter-sex variation. CKD is characterized by a decline in kidney function that is exacerbated by type 2 diabetes (T2DM), obesity, hypertension but also by inflammation and oxidative stress [5,6,7]. There is a strong association between CKD and cardiovascular disease (CVD) considering that CKD seems to be an independent risk factor for cardiovascular events and for all-cause of cardiovascular (CV) death, as it is associated with increased levels of inflammatory factors, abnormal apolipoprotein levels, elevated plasma homocysteine, enhanced coagulability, anemia, left ventricular hypertrophy, increased arterial calcification, endothelial dysfunction, and arterial stiffness [1,2,8]. On the other hand, also patients affected by primary heart diseases are more prone to develop CKD [PMID: 20037146]. Moreover, in diabetic patients renal damage is part of macro and microvascular complications that typically characterize disease progression, and contributes to increase the overall mortality rate [1,9]. For these reasons, there is a growing need to define preventive strategies that may reduce CKD incidence and slow down its progression. Lifestyle and dietary habits can influence kidney function, playing a crucial role in the prevention and development of CKD. A healthy diet decreasing CKD incidence and progression would in turn significantly decrease the burden on healthcare, with the adjunctive benefit of avoiding or reducing pharmacological intervention. Generally, dietary studies in CKD concentrated on single nutrients and foods [10,11,12]. The study of healthy dietary patterns instead, may provide a more powerful tool to evaluate the synergistic and cumulative effects of certain nutrients on CKD [13,14].

**Figure 1:**
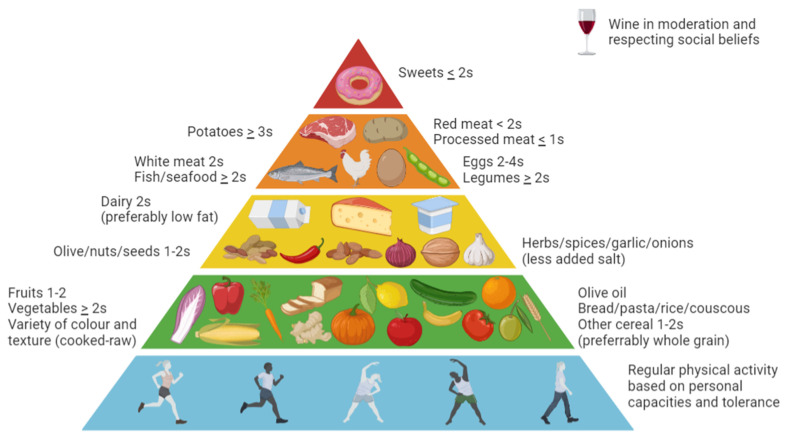
Med Diet pyramid: updated indications and guidelines for adult population. **Note:** s: servings; Adapted from “Med Diet as the diet of choice for patients with chronic kidney disease” [17].

The Med Diet (Med Diet) was first examined in medical literature in 1975 as a model of healthy eating. There is not actually a single model of Med Diet, but the main composition is modeled after the historic diet of Crete’s island [15]. This type of diet focused its nutrients intake especially on plant-based foods, including: fruits, vegetables, legumes and non-saturated fats derived from olive oil, nuts, seeds and fish. At the same time poultry, red meat and eggs were limited, but not fully excluded. Fruits were consumed especially at the end of the meal. Wine was consumed with moderation along with dairy products. Whole grain-based foods were preferred over more processed one [12,13]. Therefore, this whole food diet can lead to many physiological changes that may contribute to preserve renal health. Specifically, it can lead to improvements in blood pressure control, plasma lipid profile, systemic inflammation, body weight maintenance [10,14,15,16]. A detailed and up to date composition of Med Diet is presented in Figure 1, along with guidelines for specific nutrients intake.

The aim of the present review is to evaluate and discuss recent evidence concerning the potential benefits of Med Diet on kidney health, focusing on primary and secondary prevention of CKD.

## 2. Med Diet in Primary Prevention of CKD

Different clinical studies, both observational and interventional, have been conducted to assess the efficacy of Med Diet in preserving kidney function [18].

Most of cross-sectional and prospective studies investigated the associations between this dietary pattern and renal outcomes using scores for adherence to Med Diet [17,19,20,21]. Dietary adherence has been assessed through a diet score of up to nine most-characteristic foods groups from the pyramid that are health-promoting and/or health-harming. A semi-quantification/frequency intake of these items results in a total score (from 0 to 9) used to quantify the adherence to Med Diet pattern [22]. Therefore, we should consider that in this way, the Med Diet score is population specific. In this context, two cross-sectional studies in healthy Greek populations showed that a better observance to the Med Diet was associated with a lower albuminuria and higher creatinine clearance rate, according respectively to the Med Diet Quality Index for children and adolescents [20] and the MDS (Med Diet Score) [17,19]. Furthermore, in a cohort of 1110 Swedish elderly men, after a follow-up of 10-year it was demonstrated, according to the MDS [13,17,19], that following Mediterranean Diet was associated with a better kidney function. eGFR calculated as an estimate of serum concentrations of cystatin C (mg/L) [23]. Patients with eGFR < 60 mL/min/1.73 m^2^ were considered to have manifest CKD according to KDOQI definition [24]. Individuals with high (6–8 Mediterranean Diet Score) and medium (3–5 Mediterranean Diet Score) MDS were 42% and 23% less likely to have CKD, respectively (adjusted odds ratio [95% confidence interval] = 0.77 [0.57 to 1.05] and 0.58 [0.38 to 0.87], respectively, P for trend = 0.04).

During the ten-year follow-up, 168 (33%) CKD individuals died. Kaplan–Meier curves were used to assess survival rates in low- (≤2 points), medium- (3–5 points), and high-adherent (≥6 points) individuals. CKD Individuals with high and medium MDS adherence have a 23% and 25% lower risk of all-cause mortality, respectively [13].

More recently in an Iranian study, Asghari and colleagues analyzed the relationship between adherence to Med Diet and the 6-year incidence of CKD in 1212 adults aged 30 to 71 years. They demonstrated a significant inverse association between Med Diet and the risk of incident CKD. In particular, patients more adherent to the Med Diet pattern had a 50% lower risk of developing CKD, regardless of the presence of diabetes, hypertension, or high BMI [25]. After further adjustment for baseline eGFR, the inverse association between high MDS and incidence of CKD remained significant (OR = 0.53; 95% CI, 0.31–0.91). Furthermore, every 1-point increase in the MDS was associated with an 11% reduced risk of incident CKD at follow-up ((OR = 0.89; 95% CI, 0.80–1.00), *p* = 0.05). In the Atherosclerosis Risk in Communities (ARIC) study, involving 12,555 participants aged 45–64 years, emerged that greater adherence to healthy dietary patterns, including the Med Diet, was associated with a lower risk of developing CKD. The primary outcome was incident CKD, outlined using a composite definition: an eGFR <60 mL/min/1.73 m^2^ accompanied by ≥25% eGFR decline at a subsequent visit after baseline, and hospitalization or death related to CKD stage ≥3, or end-stage renal disease or eGFR <60 mL/min/1.73 m^2^ at a subsequent study visit accompanied by ≥30% eGFR decline relative to baseline. During the 24-year follow-up, there were 3980 cases of incident CKD. Of these cases, 56% (2237) showed a decline in eGFR. A modified adherence score for the U.S. population (aMed) assessed the adherence to the Med Diet pattern. After adjustment for BMI, diabetes, systolic blood pressure, antihypertensive medication, and HDL, patients in the highest quintile of aMED score had a 13% lower risk of incident CKD compared with those in quintile 1 [26]. Even in a German cohort [27] following Med Diet was associated with a lower prevalence of diabetes mellitus and a higher glomerular filtration rate, in contrast to what was observed in people who adhered to the diet proposed by the German Food Pyramid Index (GFPI). From single nutrients standpoint, higher eGFR was observed in people who consumed more nuts, whole grains, fish, and legumes, while higher whole-fat dairy consumption was associated with lower eGFR [25,28].

There are few dietary intervention clinical studies assessing the impact of a Med Diet on parameters associated to kidney function in a CKD prevention perspective. An earlier report from a primary prevention study, the PREDIMED (Prevención con Dieta Mediterránea) study, evaluated, in a 1-year follow-up, the impact on kidney function in elderly people without coronary heart disease (CHD) but at high risk of CVD of three different dietary interventions: two Med Diets supplemented, respectively, with EVOO and nuts and a control low-fat diet [29]. Participants randomly assigned to MedDiet groups were given free virgin olive oil (15 L every 3 months) or mixed nuts (30 g/d; 15 g/d of walnuts, 7.5 g/d of almonds, and 7.5 g/d of hazelnuts). Participants assigned to the control diet received recommendations regarding the intake reduction of all types of fats, both animal and vegetable, as recommended by the American Heart Association guidelines [30]. The authors found that eGFR improved in the 3 treatment groups compared to baseline, with no differences between dietary interventions even when the presence/absence of T2DM was considered separately. Supplemental analyses performed on PREDIMED trial cohort confirmed the positive impact of Med Diet on kidney function seen previously [31]. For this study were enrolled 5675 patients aged 55–75 with overweight or obesity (BMI ≥ 27 kg/m^2^ and <40 kg/m^2^) and free from cardiovascular disease who satisfied at least 3 criteria for the metabolic syndrome (MetS) definition. Patients at extremes of energy intake (women < 500 and >3500 kcal/die, and men < 800 and >4000 kcal/die) were excluded. Dietary intake was evaluated using food frequency questionnaires (FFQ). Some were prescribed an erMed Diet (energy-reduced Med Diet), checked with a modified energy-reduced Med Diet Score (erMDS) [32]. The other two diet regimes evaluated were DASH (Dietary Approach to Stop Hypertension) [33,34] and classic MDS [17,19]. They assessed the associations between categories of changes in dietary patterns and changes in eGFR (mL/min/1.73 m^2^). Besides, they assessed the association between categories of changes in dietary patterns and eGFR decline (≥10%). In both analyses, the decrease or maintenance of changes category was used as reference. Participants were categorized in four groups according to changes in dietary pattern scores after 1 year of follow-up: decrease or maintenance of changes and tertiles of increasing changes.

Participants in the highest tertile of increase in 17-item erMedDiet Score showed higher changes in eGFR (β: 1.87 mL/min/1.73 m2; 95% CI: 1.00–2.73) and had lower odds of ≥10% eGFR decline (OR: 0.62; 95% CI: 0.47–0.82) compared to subjects in the decrease/maintenance category, while MedDiet and DASH Scores were not associated with any outcomes regarding CKD. Not significant results were observed with the Trichopoulou-MedDiet and DASH Score. As stated by the authors, these inconsistencies could be in part explained by their differences in the score calculation, which in contrast to the 17-item erMedDiet depends on cut-off points based on the study population distribution of each item. The greatest contributors to the association between changes in erMDS [32] and eGFR changes were the consumption of ≥ 2 units/day of vegetables, ≥3 servings/week of legumes and moderate consumption of wine. Conversely, in the CORDIOPREV trial [1], the efficacy of Med Diet on the prevention of kidney dysfunction was demonstrated. In this study, 1002 coronary artery disease patients were divided into two groups. One group followed Med Diet, while the other group a low-fat diet. Renal function was assessed at baseline and after five years of dietary intervention. The results of the study showed that although eGFR decreased after both dietary interventions compared with baseline, Med Diet produced a smaller decrease in eGFR than the low-fat diet even when correcting for diabetes. So, a long-term consumption of Med Diet, rich in extra virgin olive oil and other non-saturated fats, compared with a low-fat diet, may contribute to preserve renal function.

Instead, a study that focused on Hispanic ethnicity obtained contrasting results. The HCHS/SOL (The Hispanic Community Health Study/Study of Latinos) enrolled 8711 patients, age 18–74, without baseline CKD. Diet habit was evaluated with FFQ and patients were subjected to three different diet regimes: Alternative Healthy Eating Index-2010 (AHEI-2010) [33], DASH [34] and Med Diet evaluated with MDS [13,19]. On average, eGFR declined by 0.65 mL/min/1.73 m2 per year. Lower AHEI-2010 [35] diet score was associated with greater decline in eGFR dose-response (p trend = 0.02). In contrast, DASH [34] and MDS [19] were not statistically different in regard to annualized change in eGFR. None of the three dietary prescriptions seemed to have an impact on the development of CKD [35].

## 3. Role of Med Diet in CKD Secondary Prevention

Another important point of interest is whether adherence to Med Diet could impact the progression of CKD.

Picard et al. evaluated 50 patients, aged 18–80, with diabetes mellitus and CKD stage 1–4. There were no significant differences in age, CKD stage, diabetes type and duration among patients selected [36]. The FU was of 5 years. A 3-day food record was used to assess dietary habits. Adherence to Med Diet was evaluated with MDS. In this study a lower stage of CKD was associated with higher MDS score. However, Med Diet seemed not to be significantly associated with eGFR variations during the follow-up. Higher MDS was associated with higher potassium intake, but, most notably, not with higher serum potassium concentrations. Conversely, patients with higher serum potassium levels had worse renal function and generally consumed less potassium-rich nutrients.

Another study, conducted by Moradi et al. in 270 patients with Diabetic Nephropathy (DN), produced contrasting results. The study included DN patients with serum creatinine (sCr) of 1–2.5 mg/dL, and blood urea nitrogen (BUN) of 20–40 mg/dL. Patients on a specific diet or with a daily energy intake <800 Kcal or >4200 kcal were excluded. Diet was checked by using FFQ. A trained dietitian evaluated all participants. MDS was assessed based on the eight characteristics of a traditional Med diet [17]. A maximum MDS of 9 could be obtained by summation, and participants were grouped into tertiles based on Med diet score. In this research, eGFR, sCr and BUN seemed to be not associated with Med Diet, either in crude or multivariable-adjusted models [37].

On the other hand, a very recent work, conducted within the frames of the ongoing CORDIOPREV study (a prospective RCT) found very interesting results [1]. This study enrolled 1002 patients with established coronary heart disease, but without events in the past 6 months and without any other serious illnesses. Patients were then randomized to two different diets: Med Diet and a low-fat diet (in accordance with the recommendations of the National Cholesterol Education Program). No interventions to increase physical activity or aimed to obtain weight loss were made. The evaluation of the patients lasted 5 years. Estimated GFR declined in both groups after dietary intervention but the rate of eGFR loss was significantly lower in patients who were randomized to Med Diet (*p* = 0.033). When considering baseline eGFR, this effect remained significant only among patients with eGFR between 60–90 mL/min. In particular, eGFR decline rate was 2.49 mL/min/1.73 m^2^ lower in Med Diet group compared to those that were prescribed a low-fat diet (*p* = 0.040). No changes in albuminuria were observed after any of the two dietary interventions. Moreover, Med Diet produced a significantly lower decay of renal function also in diabetic patients (eGFR decline rate was 2.07 mL/min/1.73 m^2^ lower compared to the low-fat diet). However, the most important result was the independent association of Med Diet with eGFR changes even after correction for baseline eGFR, diabetes and age ((B = 1.474, *p* = 0.041).

Heindel et al. in a cohort of 2813 patients [27], obtained similar results. This prospective observational cohort study enrolled patients with CKD stage 1–3. Dietary habits were collected using FFQ derived from the European Prospective Investigation into Cancer and Nutrition (EPIC) study [38]. In this cohort 3 different dietary patterns were evaluated: DASH diet, [34] Med Diet [19] and GFPI [39] (German Food Pyramid Recommendation). In particular, to evaluate Med Diet, a mMDS (modified MDS) for non-Mediterranean population was used [17]. In this study every SD (standard deviation) increase of mMDS was directly associated with higher eGFR (β = 0.932, *p* = 0.007), even after adjustment for significant risk factors as: gender, age, BMI, caloric intake, smoking status, alcohol consumption, school education, HDL-cholesterol, LDL-cholesterol, hypertension and diabetes. In particular, high intakes of cereals (β = 1.733, *p* = 0.020), fish (β = 1.630, *p* = 0.017), and unsaturated fats (β = 1.755, *p* = 0.011) were associated with higher eGFR. Only higher intake of dairy products (low and whole-fat dairy) was associated with lower eGFR (β = 21.633, *p* = 0.018).

Med Diet seems to play a favorable effect on renal outcomes even in patients who underwent renal transplantation. Gomes et al. evaluated 632 patients with renal transplantation [40]. The primary end point was graft failure, defined as return to dialysis or re-transplantation. Secondary end points were the composite end points defined as: doubling of serum creatinine or graft failure, and graft loss defined as graft failure or death with a functioning graft. No specific dietary counselling prior the beginning of the trial was given, except for lowering sodium intake and to lose weight if a patient had high BMI. No peri-transplant complications were present in patients of this cohort. Diet intake was assessed through the validated FFQ and adherence to Med Diet was evaluated through the MDS, median follow-up was 5.4 years. MDS was inversely associated with graft failure (HR: 0.76–95% CI: 0.58 to 0.98—5-year absolute risk reduction (ARR), 3%), kidney function decline (HR: 0.76–95% CI: 0.62 to 0.93—5-year ARR, 4%), and graft loss (HR: 0.78–95% CI: 0.66 to 0.92—5-year ARR, 5%). These associations were still significant after adjustment for age, sex, BSA, eGFR, urinary protein excretion, time since transplantation, primary kidney disease, HLA mismatches, living versus deceased donor kidney, and preemptive transplantation (graft failure: HR: 0.68–95% CI: 0.50 to 0.91—kidney function decline: HR: 0.68–95% CI, 0.55 to 0.85—and graft loss: HR: 0.74–95% CI: 0.63 to 0.88). These associations remained valid even after further correction for immunosuppressive treatment, cardiovascular risk factors, and lifestyle factors. Higher intake of legumes and nuts was inversely associated with graft failure (HR: 0.57–95% CI, 0.36 to 0.91) and higher than median intake of cereals (HR: 0.66–95% CI, 0.49 to 0.89) and moderate alcohol intake (HR: 0.63–95% CI: 0.44 to 0.89) were both inversely associated with graft loss. Finally higher MDS was associated with lower risk of graft failure especially in patients with higher proteinuria and shorter transplant time.

## 4. Med Diet and Metabolic Complications in Patients with CKD

In patients with CKD, Med Diet has several metabolic effects that deserve to be analyzed. Patients with CKD, especially those in more advanced stages, are at high risk of hyperkalemia [41], which is associated with increased mortality [42]. Med Diet, like other healthy diets, contains a significant amount of foods with an high potassium content [43] such as whole grains, fruits and vegetables [44]. In addition, it is worth noting, that also dairy products, animal proteins and beverages contribute significantly to dietary potassium intake [45] but their consumption is limited in Med Diet. Picard et al. [36] in their post-hoc analysis of a RCT assessed the relationship between Med Diet and potassium intake in CKD patients (study where diets were sorted for potassium intake above 2000 mg/day vs. below 2000 mg/day). Of the 50 participants, 9 had hyperkalemia. Five participants had hyperkalemia once, four had hyperkalemia more than once, for a total of 15 events. No association between dietary potassium intake and serum potassium levels was found. Interestingly, they found that those with better diet quality consumed more potassium than those with poor diet quality but did not have higher rates of hyperkalemia or higher serum potassium levels. In another study Heindel et al. [27] evaluated three dietary patterns rich in potassium: Med Diet, DASH and a diet proposed by the German Food Pyramid Index. Their data suggest that these three dietary patterns could be safe since they didn’t observe any hyperkalemia in their patients. Lack of correlation between potassium from whole foods and serum levels can be explained with the lower bioavailability of potassium in unprocessed and minimally processed foods. In fact, when plant cell walls remain intact, this could blunt the effect on serum potassium levels [46]. Additionally, it has been proposed that plant foods, that are base-inducing foods, tend to shift potassium intra-cellularly minimizing the impact on serum levels [47]. Thereof, we may summarize that although Med Diet is generally characterized by a high content of potassium there are not evidences suggesting that in CKD patients it may be associated with higher incidence of hyperkalemia. However, it is noteworthy that none of these studies was performed in subjects with advanced CKD where a severe reduction of eGFR may interfere with urinary potassium excretion and may induce potassium accumulation and hyperkalaemia.

Med Diet is rich in monounsaturated fats (MUFA) from olive oil (mainly VOO or EVOO) and poor in saturated fats (SFA) from meat and dairy products. For this reason, Med Diet is widely recognized for its benefits in reducing cardiovascular risk factors by improving lipid profile [1]. Moradi et al. in their cross-sectional study measured serum lipids, total cholesterol (TC), low-density lipoprotein cholesterol (LDL-C) and triacylglycerols (TG) in 270 patients with diabetic nephropathy stage 1–2. A trained dietitian evaluated all participants. Med Diet score was assessed on basis of the eight characteristics of a traditional Med Diet that were outlined by Trichopoulo et al. [21]. Participants were given one point for: daily portion of fruit, fish, vegetables, whole grains, legumes or nuts, if the ratio of MUFA to SFA was equivalent to or greater than the median intake of the study population, and if the daily portion of meat (red meat, poultry and processed meats) and dairy products were less than the median intake of the study population. They found that lipid profile was not significantly associated with Med Diet observance in either crude or multivariate-adjusted models. Also Picard et al. and in the CORDIOPREV study did not find any association between diet quality and TG, LDL or HDL [1,36].

Thanks to the presence of high-quality carbohydrates provided by whole grains, nuts, fruits, and vegetables [22] Med Diet is characterized by a low glycemic index, with positive effects on glucose control, hyperinsulinemia, insulin response and decreased appetite. Furthermore, Med Diet provides 30 to 50 g of fibers per day [22,48] that can contribute to improve postprandial glycaemia and to improve insulin sensitivity. Heindel et al. [27] also in a cohort of 2813 German CKD patients, aged over 60 years, confirmed these evidences. High Med Diet score was associated with lower incidence of diabetes and lower HbA1c in contrast to what was observed in people who adhered to the diet proposed by the German food pyramid (GFPI) [27]. This is consistent with the results found in another large German prospective cohort study in which better adherence to Med Diet was associated with lower prevalence of diabetes mellitus in the general population [49]. In a small observational study [50] conducted in 99 Australian CKD patients (stages 3 to 5), adherence to Med Diet was associated with an improved glycemia as well as with a lower incidence of type 2 diabetes.

As for other potential benefits of Med Diet on hyperphosphatemia and metabolic acidosis in CKD patients, we did not find any study specifically addressing these aspects. See Table 1 for reviewed studies.

## 5. Conclusions

From the data presented in this review the potential beneficial effect of Med Diet on CKD seems evident, both in terms of prevention of the development of established disease as well as in terms of slowing disease progression. In particular, positive renal outcomes seem to be more dependent on the consumption of specific nutrients, as legumes, nuts and vegetables. Conversely, red/processed meat and high alcohol intake had in general a negative impact on renal health. Furthermore, Med Diet seems to have a positive effect also on metabolic control of diabetic patients, could help to reduce the incidence of renal and cardiovascular complications in this high-risk population.

However, further studies are needed, especially to clarify the effectiveness of Med Diet in slowing CKD progression, and the impact of ethnicity and previous dietetic regimes on Med Diet efficacy. In fact, a relevant limitation that emerged from our review is that very few studies have evaluated the benefits of the Med Diet in slowing the progression of disease especially in advanced stages of CKD. Moreover, it would be interesting to evaluate the influence of Med Diet on the evolution of CKD based on different primary renal diseases and/or in the presence of different comorbidities.

## Figures and Tables

**Table 1:** Summary of principal studies and paper evaluating Med diet impact on kidney function.

Study	Type	Methods	Results
Primary prevention studies			
Adherence Med Diet and albuminuria levels in Greek adolescents: data from the Leontio Lyceum ALbuminuria (3L study). Mazaraki et al. [20]	Longitudinal prospective cohort study	365 healthy adolescents aged 12–17. Adherence to Med Diet was assessed using the KIDMED score [25].	Lower adherence to Med Diet was significantly and independently associated with higher ACR.
Adherence Med Diet is Associated With Renal Function Among Healthy Adults: The ATTICA Study. Chrysohoou et al. [19]	Longitudinal epidemiological cohort study	3042 healthy patients, age 18–88. 1 year FU. Diet was evaluated using EPIC score [36]. Adherence to Med diet was evaluated with MDS [9]	Creatinine clearance was directly and independently associated with higher MDS. In unadjusted analyses urea and creatinine were inversely associated with MDS.
Med Diet, Kidney Function, and Mortality in Men with CKD. Huang et al. [13]	Longitudinal prospective cohort study (based on ULSAM)	1110 male patients, average age 70 years, with and without baseline CKD. 10 years FU. Dietary habits evaluated with SNFA [40]. Adherence to Med Diet was evaluated with adapted MDS [9].	Higher MDS was significantly and independently associated with lower OR of developing CKD. Moreover, higher MDS was associated to significantly better survival in patients with CKD.
Adherence to Med Diet is associated with reduced risk of incident chronic kidney diseases among Tehranian adults. Ashgari et al. [25]	Longitudinal prospective cohort study (based on TLGS)	1212 patients (age 30–70). Patients with CKD, previous MI, stroke, cancer or <800 Kcal/die/>4200 Kcal/die were excluded. Diet was assessed using FFQ, adherence to Med Diet was assessed with MDS [9].	Higher MDS was significantly and independently associated with a lower risk of CKD development, even after adjustment for eGFR.
Dietary patterns and risk of incident chronic kidney disease: the Atherosclerosis Risk in Communities study. Hu et al. [26]	Longitudinal prospective cohort study (based on ARIC)	12155 patients (age 45–64). Patients with CKD at baseline, history of MI, cancer or females with <500 or >3500 kcal/die or males with <700 or >4500 kcal/die were excluded. Diet was assessed with FFQ. Diet adherence was assessed with HEI-2015 [17], AHEI-2010 [28] and aMed [23] (modified from standard MDS). Median FU 24 yrs.	Adherence to Med Diet was significantly and independently associated with lower risk of incident CKD. In particular nuts, whole grain and moderate alcohol consumption were associated with a lower risk of CKD development, while red/processed meat was associated with higher risk of CKD.
Effects of Med Diets on Kidney Function: A Report From the PREDIMED Trial. Diaz-Lopez et al. [29]	Longitudinal prospective RCT	665 Patients aged 55–80. Patients with history of CVD, severe chronic illness, substance addiction were excluded. 1 year FU. 3 types of diet: Med Diet + olive oil, Med Diet + nuts, control low fat diet.	eGFR increased significatively in all 3 groups. No significant between group difference in eGFR and albuminuria progression was noted.
Association of Diet Quality Indices with Longitudinal Changes in Kidney Function in U.S. Hispanics/Latinos: Findings from the Hispanic Community Health Study/Study of Latinos (HCHS/SOL) Missikpode et al. [35]	Prospective observational cohort study	8771 patients from the HCHS/SOL study [22,23]. Median FU 6 years. Healthy diet regime adherence evaluated with AHEI-2010 [29], DASH [28], MDS [9]. Patients with CKD at baseline were excluded.	Lowest AHEI-2010 quartiles were associated with greater decline in eGFR. In particular, higher consumption of whole fruit was associated with a slower annual decrease in eGFR. DASH and Med Diet quartiles were not significantly correlated with annualized eGFR variation. None of 3 dietetic patterns were associated with incident CKD
Prospective associations between a priori dietary patterns adherence and kidney function in an elderly Mediterranean population at high cardiovascular risk Valle-Hita et al. [31]	Prospective observational cohort study	5675 patients age 55–75, overweight or obese, from PREDIMED-Plus study [27]. Patients excluded if extreme low/high energy intake (<500 Kcal/die w./<800 Kca/die m. - >3500 Kcal/die w./>4000 Kcal/die m.). Diet evaluated using FFQ. Energy reduced Med Diet was evaluated using erMDS [26] classic MDS [9]. Diet regimes also evaluated with DASH [29]. 1 year FU.	Adherence to erMed Diet was significantly and independently associated with lower decrease in eGFR at 1 year, in a dose dependent fashion. The same was true when considering the OR of 10% eGFR decline. Nutrients mostly associated with eGFR changes were vegetables, legumes, and moderate wine consumption. No significant association were found for classic Med Diet [19] or DASH [17].
Recent secondary prevention studies			
Med Diet and Kidney Function Loss in Kidney Transplant Recipients. Gomes et al. [40]	Longitudinal cohort study	632 Rtx-p with a functioning graft for ≥1-year. Standard IS and overall therapy, no prior specific dietary counseling. Diet evaluated using FFQ, Med Diet evaluated using Med Diet Score (MDS) [22] using a 9-point scale	MDS was inversely associated with graft failure (HR 0.76; 5 yrs ARR 3%), kidney function decline (HR 0.76; 5 yrs ARR 4%) and graft loss (HR 0.78; 5 yrs ARR 5%) independently from confounders. MDS also associated with longer time to graft failure, especially if high Prot-U was present.
Low Med Diet scores are associated with reduced kidney function and health related quality of life but not other markers of cardiovascular risk in adults with diabetes and chronic kidney disease. Picard et al. [36]	Post-Hoc analysis of a RCT longitudinal study	50 patients with diabetes and CKD stage 1–4, age from 18–80. 5-year FU. 3-day food record was used to assess nutrients intake. MDS calculated with a 9-point scale [22].	Stage 1–2 vs. Stage 3–5 CKD was associated with higher MDS, but no association of MDS in and eGFR decline rate. Higher K^+^ intake was associated to higher MDS but not to higher serum K^+^. Hyperkalemic patients had lower K^+^ intake and worse eGFR.
Association between adherence to the Med Diet and renal function biomarkers and cardiovascular risk factors among diabetic patients with nephropathy. Moradi et al. [37]	Cross-sectional study	270 patients with DN, Prot-U 300–1000 mg/dL, sCr 1–2.5 mg/dL. Diet evaluated using FFQ. MDS was calculated with a 9-point scale [22]. Patients with daily intake <800 Kcal or >4200 Kcal were excluded.	sCr, BUN, eGFR, CRP not significantly associate to Med diet adherence. No relevant association also with CVD markers.
Long-term consumption of a Med Diet or a low-fat diet on kidney function in coronary heart disease patients: The CORDIOPREV randomized controlled trial. Podadera-Herreros et al. [1]	RCT	1002 CHD patients, last CV event >6 mths before enrolling, age 20–75. Randomization based on age, sex and previous MI between Med diet and low-fat high complex carbohydrate diet according to (National Cholesterol Education Program). 5 yrs FU.	eGFR declined significantly after both dietary interventions, Med Diet was independently (vs baseline eGFR, sex, DM) and significantly associated with a lower eGFR decline both in DM and non-DM patients, especially in patients with baseline eGFR between 60–90 mL/min
Association Between Dietary Patterns and Kidney Function in Patients With Chronic Kidney Disease: A Cross-Sectional Analysis of the German Chronic Kidney Disease Study Heindel et al. [27]	Prospective observational cohort study	2813 all-cause CKD patients, stage 1–3, or stage 1–2 with overt Prot-U. 2 years FU. Diet evaluated using FFQ adapted from EPIC study. 3 different diets: DASH [28], Med diet and GFPI. Med Diet evaluated trough modified MDS [8].	Med Diet was independently associated with higher eGFR. In particular, higher intake of cereals, fish, unsaturated fats were significantly associated with higher eGFR, while dairy products were associated with lower eGFR.

**Note:** ACR: albumin to creatinine ratio; EPIC: European Prospective Investigation into Cancer and Nutrition; ULSAM: Uppsala Longitudinal Study of Adult Men; SNFA: Swedish National Food Administration; TGLS: Teheran Glucose and Lipid Study; ARIC: Arteriosclerosis Risk In Communities; HEI: Healthy Eating Index; RTx-p: renal transplant patients; IS: immunosuppressive therapy; FFQ: Food frequency questionnaire; MDS: Med Diet score; HR: hazard ratio; ARR: absolute risk ratio; Prot-U: urinary protein excretion in 24 h; RCT: randomized controlled trial; CKD: chronic kidney disease; FU: follow-up; eGFR: estimated glomerular filtration rate; DN: diabetic nephropathy; BUN: blood urea nitrogen; CRP: c-reactive protein; sCr: serum creatinine; CVD: cardiovascular disease; CV: cardiovascular; MI: myocardial infarction; DM: diabetes mellitus; DASH: dietary approach to stop hypertension; GFPI: german food pyramid index; AHEI: alternate healthy eating index; erMDS: reduced energy Med Diet score.

## Data Availability

Not applicable.
